# Supplementary material for: Tumor Doubling Time Using CT Volumetric Segmentation in Metastatic Adrenocortical Carcinoma
Source: Curr Oncol. 2021 Nov 1;28(6):4357–66. doi: 10.3390/curroncol28060370 (PMC8628706; doi:10.3390/curroncol28060370)
Supplement: Supplementary file 1 [file curroncol-28-00370-s001.zip › curroncol-1345332-supplementary.pdf]

*Supplementary Materials*

# Tumor Doubling Time Using CT Volumetric Segmentation in Metastatic Adrenocortical Carcinoma

**Sarah N. Fuller, Ahmad Shafiei, David J. Venzon, David J. Liewehr, Michal Mauda Havanuk, Maran G. Ilanchezhian, Maureen Edgerly, Victoria L. Anderson, Elliot B. Levy, Choung D. Hoang, Elizabeth C. Jones, Karlyne M. Reilly, Brigitte C. Widemann, Bradford J. Wood, Hadi Bagheri and Jaydira Del Rivero**

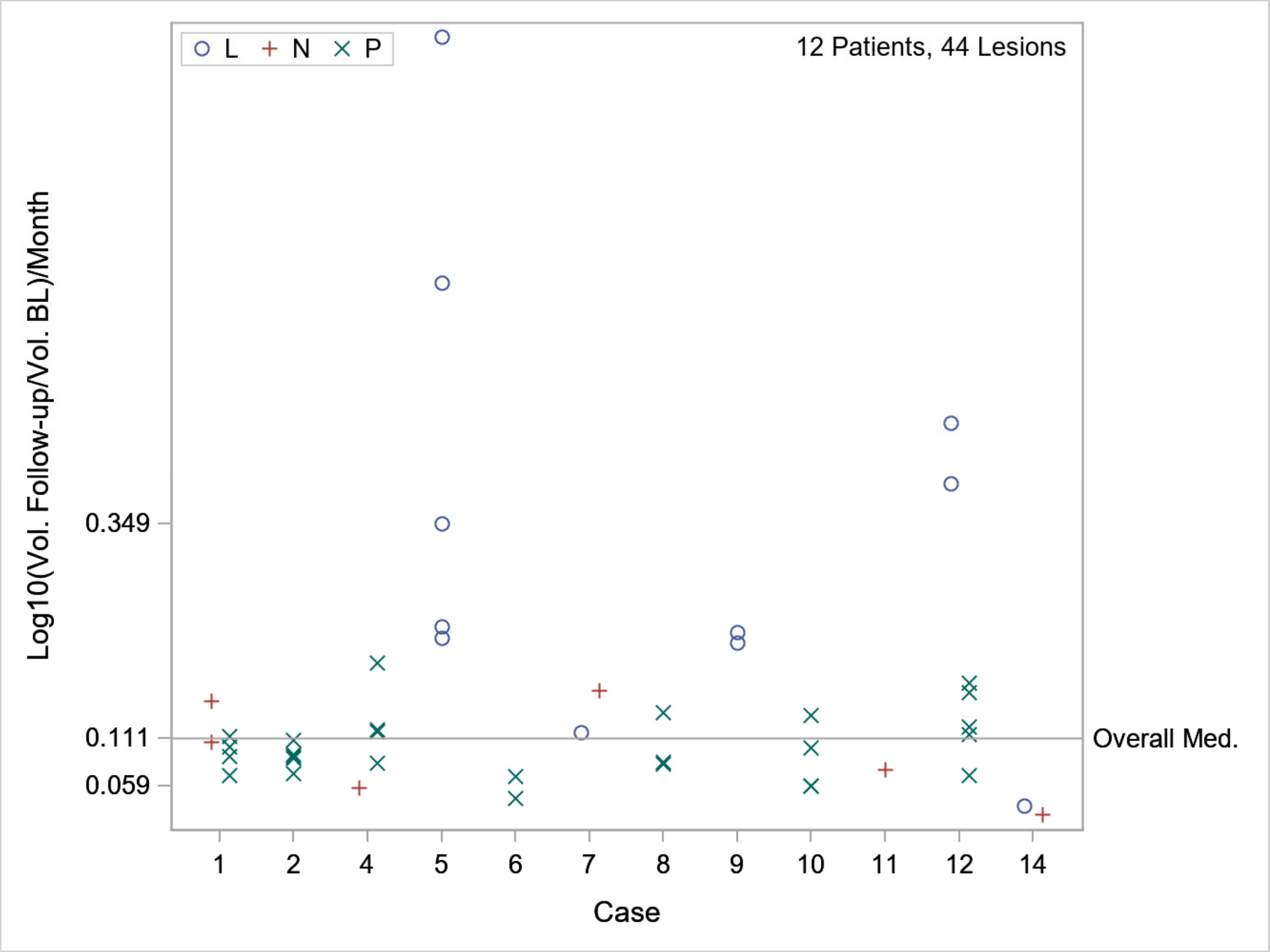


**Figure S1.** Scatter plot of exponential growth rate vs. case number (patient ID, Table 1) by type of lesion (L=Liver, N=Lymph Node, P=Pulmonary (Lungs)). Besides the overall median, the other two y-axis values indicate the 10th and 90th percentiles. Note the large growth rates for the liver lesions for patients 5 and 12, and the small liver lesion growth rate for patient 14.

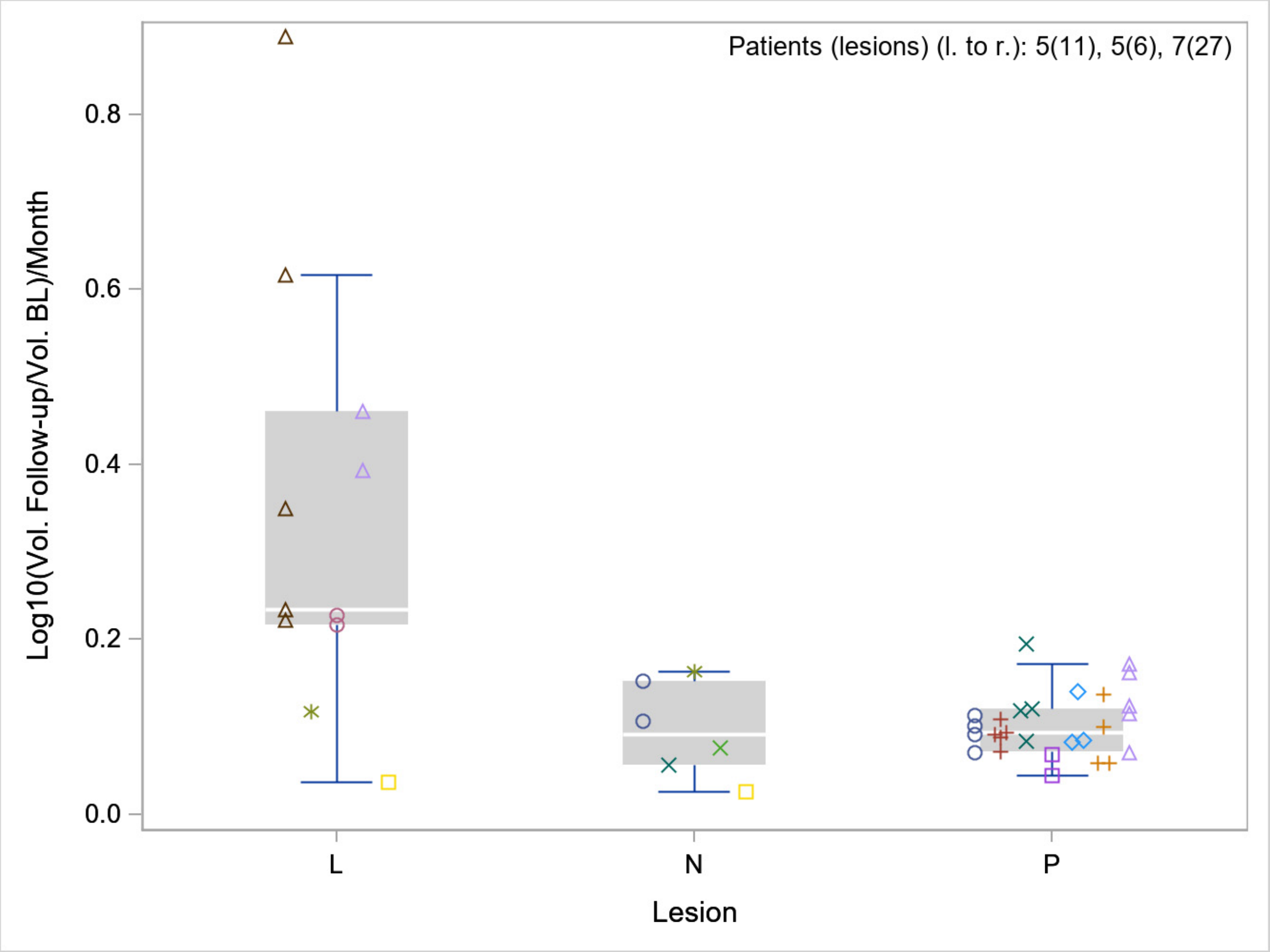


**Figure S2.** Box-and-whisker plots of exponential growth rates by lesion location. Each combination of symbol and color represents a patient. Note the extreme outlier for lesion L.

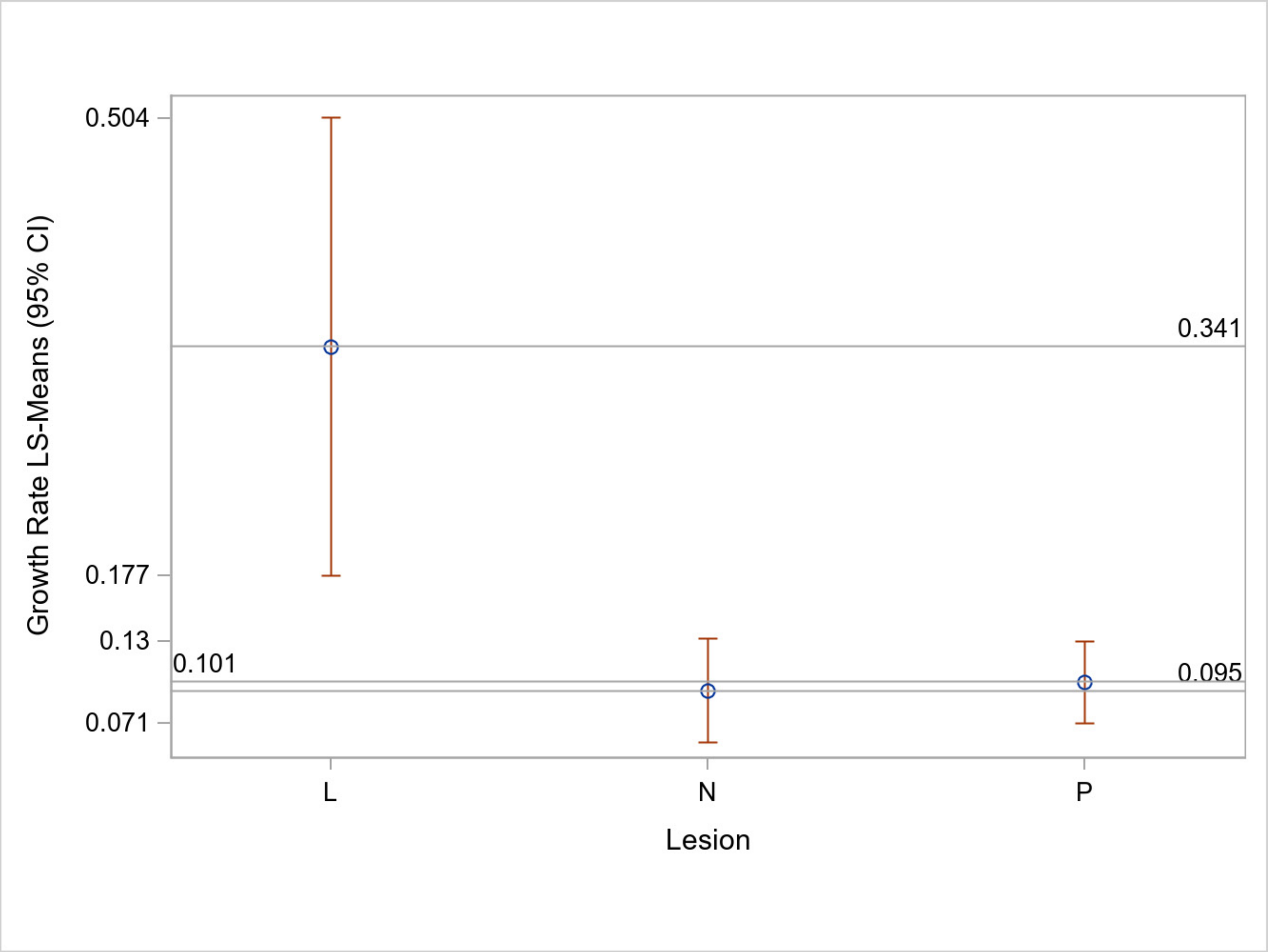


**Figure S3.** Mixed model LS-Means (reference lines) and their 95% CIs. Y-axis values indicate approximate CI limits.

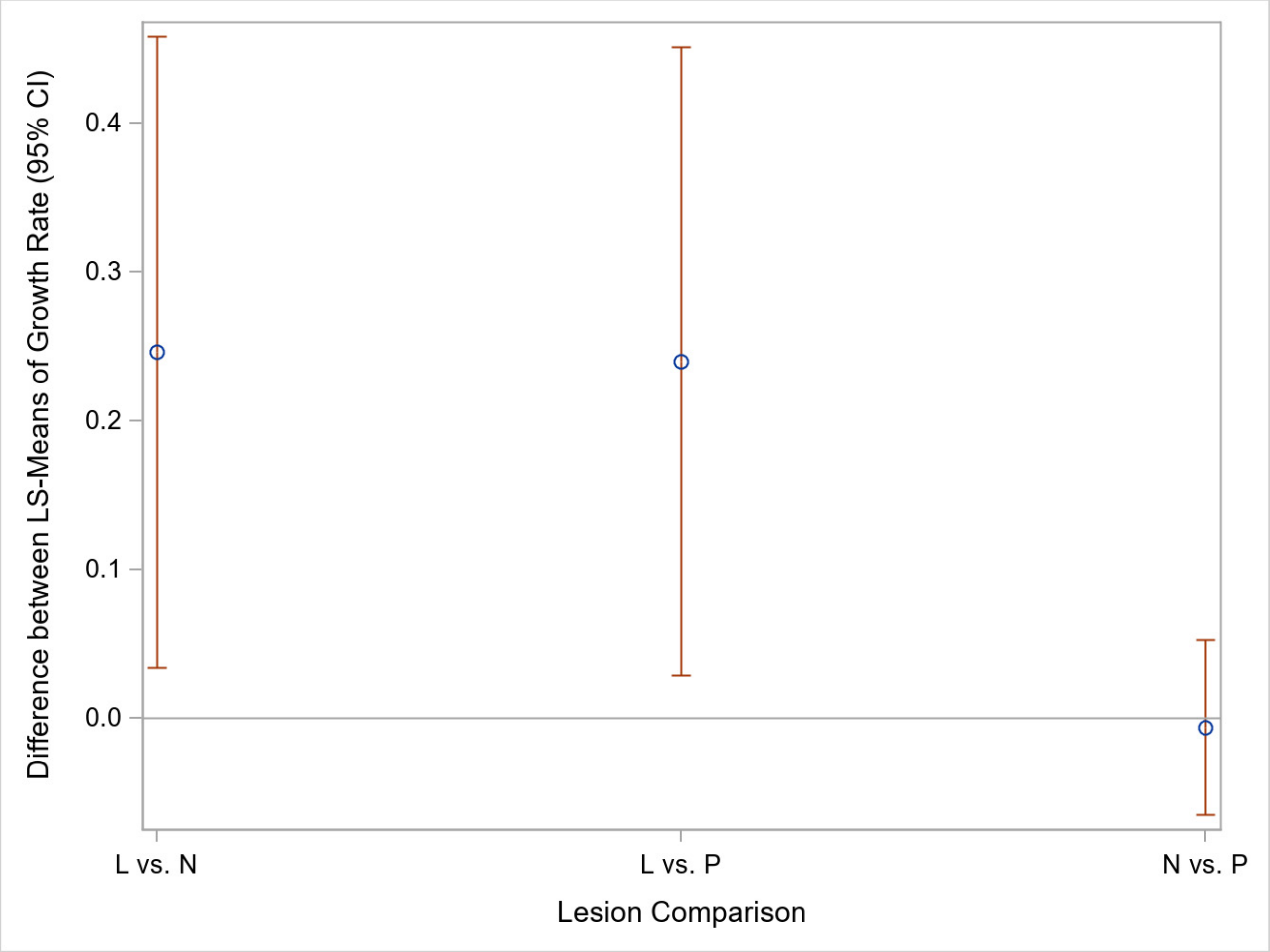


**Figure S4.** Differences between LS-Means and their 95% CIs (Bonferroni adjusted). Note that an increase of 0.1 in the N or P growth rate would increase the corresponding volume doubling time by a factor of 2.

**Doubling Time Suppl. Stat. Section**

The initial data analysis of this exploratory study was performed on growth rates from 14 patients (cases) who had 56 lesions (Table 1), each classified in one of three lesion locations (L=liver, N=lymph node, P=lung) excluding measured lesions in other locations (n=2). However, two patients (cases 3 and 13) were subsequently dropped from the analysis as they were ineligible. The final data analysis was performed on 12 patients with 44 lesions.

The data structure can be described as a generalized unrandomized unbalanced incomplete block design. It is generalized in that there are repeats of the same lesion location within a patient; the number of lesions per type per patient varies from 0 to 5. Clearly, the lesions were not randomized to the patients; nor were the patients randomly selected (potential bias). As Table 1 indicates, the number of lesions is unbalanced in that the column totals are unequal. The many zeros indicate the incomplete data structure. Most patients (7/12) had only one lesion location, whereas 5/12 patients had two locations, and none had three.

**Table S1. Case by Location.**

| Case | Location | | | |
| --- | --- | --- | --- | --- |
| Frequency | L | *N* | P | Total |
| 1 | 0 | 2 | 4 | 6 |
| 2 | 0 | 0 | 5 | 5 |
| 3* | 4 | 1 | 5 | 10 |
| 4 | 0 | 1 | 4 | 5 |
| 5 | 5 | 0 | 0 | 5 |

| | | | | |
|---|---|---|---|---|
| 6 | 0 | 0 | 2 | 2 |
| 7 | 1 | 1 | 0 | 2 |
| 8 | 0 | 0 | 3 | 3 |
| 9 | 2 | 0 | 0 | 2 |
| 10 | 0 | 0 | 4 | 4 |
| 11 | 0 | 1 | 0 | 1 |
| 12 | 2 | 0 | 5 | 7 |
| 13* | 0 | 1 | 1 | 2 |
| 14 | 1 | 1 | 0 | 2 |
| Total | 15 | 8 | 33 | 56 |
| Total after exclusion of indicated rows | 11 | 6 | 27 | 44 |

* Excluded from final analysis.

**Data Analyses**

Analysis of variance (ANOVA)

Various volume-derived outcomes were initially analyzed in an exploratory fashion: volume doubling time, diameter rate of change per month (both based on volumetric segmentation), and percent difference from baseline (RECIST). All three outcomes were analyzed similarly to the reported growth rate outcome. Results may be reported elsewhere.

The lesion growth rate was calculated using the formula:

GR = $Log_{10}$(volume at follow-up/volume at baseline)/(Months between scans).

The implied monthly growth factor is therefore $10^{GR}$, and the doubling time in days is 30.4x$Log_{10}(2)/GR$.

As stated in the manuscript, the lesion location (liver, lymph node or lung) was specified as a fixed effect in the mixed model analysis of variance, and patient by location was specified as the random effect. For the lesion effect only model, the null hypothesis of equal rates was assessed by an F-test on 2 and 10.7 numerator and denominator degrees of freedom (slightly varied with other effects in the model).

**Table S2.** Mixed model ANOVA results.

| Model* | Effect | Num Df | Den Df | F | P | Slope Est. (95% CI) |
|---|---|---|---|---|---|---|
| 1 | Location | 2 | 10.7 | 5.32 | 0.025 | |
| 2 | Location | 2 | 9.66 | 5.34 | 0.027 | |
| | Sex | 1 | 5.27 | 0.2 | 0.67 | |
| 3 | Location | 2 | 10.7 | 5.39 | 0.024 | |
| | Age | 1 | 6.45 | 0.84 | 0.39 | -0.00073 (-0.00266, 0.00119) |
| 4 | Location | 2 | 11.9 | 6.2 | 0.014 | |
| | Burden | 1 | 7.31 | 6.9 | 0.033 | 0.0117 (0.00125, 0.0221) |

* Each model also contained a patient by locaton, and residual random effect

Non-parametric summary statistics for lesion growth rate. Note that the median for location L is much less than the model-based LS-Mean of 0.341.

| | | Log10(Vol. Follow-up/Vol. BL)/Month | | | | |
|---|---|---|---|---|---|---|
| *Location* | *N* | *10th Pctl* | *25th Pctl* | *50th Pctl* | *75th Pctl* | *90th Pctl* |
| L | 11 | 0.118 | 0.217 | 0.235 | 0.461 | 0.617 |

| | | | | | | |
|---|---|---|---|---|---|---|
| N | 6 | | 0.057 | 0.092 | 0.153 | |
| P | 27 | 0.059 | 0.072 | 0.093 | 0.121 | 0.162 |

**Correlation analysis**

Various non-parametric (Spearman's) correlation analyses were performed as well, but none of the results are reported here.
